# Acaricide resistance in *Amblyomma cajennense* s.l. and implications for integrated tick control in Ecuadorian cattle systems

**DOI:** 10.3389/fvets.2026.1873156

**Published:** 2026-08-27

**Authors:** Ximena Pérez-Otáñez, Sophie O. Vanwambeke, Valeria Paucar-Quishpe, Cecilia Pérez, Jorge Grijalva-Olmedo, Darío Cepeda-Bastidas, Geoconda Orozco, Sandra Enríquez Morillo, Claude Saegerman, Lenin Ron-Garrido, Richar Rodríguez-Hidalgo

**Affiliations:** 1 Facultad de Medicina Veterinaria y Agronomía, Universidad UTE, Quito, Ecuador; 2 Grupo de Investigación CABSAC, Facultad de Medicina Veterinaria y Agronomía, Universidad UTE, Quito, Ecuador; 3 Instituto de Investigación en Zoonosis (CIZ), Universidad Central del Ecuador, Quito, Ecuador; 4 Georges Lemaitre Centre for Earth and Climate Research, Earth and Life Institute, UCLouvain, Louvain-la-Neuve, Belgium; 5 Facultad de Medicina Veterinaria y Zootecnia, Universidad Central del Ecuador, Quito, Ecuador; 6 Facultad de Ciencias Agrícolas, Universidad Central del Ecuador, Quito, Ecuador; 7 Research Unit of Epidemiology, Risk Analysis and Biosecurity applied to Veterinary Science (UREAR-ULiège), Fundamental and Applied Research for Animals and Health (FARAH) Center/Faculty of Veterinary Medicine, University of Liege, Liège, Belgium

**Keywords:** acaricide resistance, *Amblyomma cajennense*, cattle, Ecuador, three-host-tick

## Abstract

**Introduction:**

*Amblyomma cajennense sensu lato* represents a significant challenge for sustainable tick control in tropical cattle systems due to its ecological adaptability, wide host range, and frequent co-occurrence with *Rhipicephalus microplus*. This study evaluated the occurrence of acaricide resistance in *A. cajennense* s.l. populations collected from cattle farms in coastal Ecuador.

**Methods:**

A cross-sectional field assessment was conducted on seven farms (Esmeraldas, *n* = 2; Manabí, *n* = 5) that met the minimum requirement of 30 engorged *A. cajennense* s.l. females per farm for resistance testing. Tick larvae were subjected to larval packet tests to determine susceptibility to three commonly used acaricides: amitraz, ivermectin, and alpha-cypermethrin.

**Results:**

Phenotypic resistance to at least one acaricide was detected on all evaluated farms. Resistance prevalence was 43% for amitraz, 86% for ivermectin, and 100% for alpha-cypermethrin. Multi-resistance occurred on 86% of farms. Estimated lethal dose values revealed extremely high LD₉₀ and LD₉₉ levels, frequently exceeding recommended commercial concentrations by several orders of magnitude. Exploratory screening suggested that extensive cattle production systems and the presence of alternative hosts, particularly horses and other equids, may contribute to resistance emergence and persistence.

**Discussion:**

The results are particularly relevant because *A. cajennense* s.l. frequently co-occurs with *R. microplus* on cattle farms, although the two species differ substantially in ecology, host use, and life cycle. Control strategies primarily designed for *R. microplus*, such as whole-animal acaricide treatments, may have limited impact on *Amblyomma* populations maintained through wildlife hosts and environmental stages. This study provides the first evidence of acaricide resistance in *A. cajennense* s.l. in Ecuador and highlights the need for species-specific management strategies, improved resistance monitoring, and integrated tick control programs adapted to multi-species infestations in tropical livestock systems.

## 1 Introduction

The *Amblyomma cajennense sensu lato* is a three-host tick with broad host plasticity, primarily infesting equines but opportunistically parasitizing cattle, wild animals, and humans (1). This is a species complex (comprising *A. cajennense, A. interandinum, A. mixtum, A. patinoi, A. sculptum,* and *A. tonelliae*) that significantly affects public health, animal welfare, and livestock productivity across tropical and subtropical regions (2). As part of the genus *Amblyomma*, which encompasses nearly 130 species globally (3), the complex is a critical vector of diverse pathogens including protozoa, spirochetes, rickettsia, and viruses (4). For instance, in ruminants, members of this genus transmit *Ehrlichia ruminatium,* the obligate intracellular bacterium that causes heart water (cowdriosis), a disease well-documented in sub-Saharan Africa and in the Caribbean (5); however, its presence in Ecuador and other South America countries is not well documented (6).

Due to its broad host range and high ecological plasticity, *A. cajennense* is able to exploit a wide variety of vertebrate hosts across different developmental stages. This adaptability facilitates its wide distribution across the Americas, from the southwestern United States to northern Argentina, including Ecuadorian tropical regions, where it threaten livestock health and productivity by causing anemia and physiological stress associated with blood-feeding, ultimately reducing weight gain and milk production in cattle (7).

*Amblyomma cajennense* s.l. (*A. cajennense* s.l.) represents a critical challenge for integrated tick control in cattle-producing regions such as coastal Ecuador, where favorable bioclimatic conditions allow its persistence alongside *Rhipicephalus microplus*. The co-occurrence of these two tick species complicates control strategies because they differ markedly in ecology, host use, and susceptibility to acaricides. For example, control measures targeting *R. microplus* (e.g., whole-animal acaricide treatments) have limited impact on *Amblyomma* populations, which are maintained through equines and, to a lesser extent, wildlife. In addition, each tick species transmits distinct pathogens; therefore, inadequate control of one species may sustain pathogen circulation, rendering integrated disease management more complex and less predictable.

While *A. cajennense* s.l. is not a primary disease vector in Ecuador, its economic impact on cattle, through reduced weight gain and hide damage, is substantial. Unlike the one-host tick *R. microplus,* its three-host life cycle involves prolonged off-host periods and multiple host species, rendering conventional control strategies ineffective. In this context, accurate field-based assessment of resistance levels in *A. cajennense* s.l. is essential for designing targeted management strategies that balance efficacy and sustainability. Regular monitoring allows the identification of resistance trends, supports evidence-base practices such as acaricide rotation, dose optimization, and the integration of alternative methods (8, 9).

Chemical control through the use of acaricides remains the primary tick-control method available to cattle producers. As a consequence, farmers often rely on intensified chemical control, which increases the risk of acaricide overuse, elevates selection pressure, and promotes the development of cross-resistance (10). The emergence of acaricide resistance reduces the effectiveness of conventional compounds, leading to increased treatment costs, production losses, and a heightened risk of pathogen transmission (4, 11).

Given the widespread occurrence of acaricide resistance in *R. microplus* in Ecuador (6, 12, 13), together with the documented co-occurrence of both species on cattle (14), we hypothesized that *A. cajennense* s.l. may also exhibit resistance to commonly used acaricides. In this study, we quantified resistance levels to amitraz, ivermectin, and alpha-cypermethrin. Also we aimed to determine whether *A. cajennense* s.l. populations in Ecuador have developed resistance to amitraz, ivermectin, and alpha-cypermethrin, and to identify farm-level factors associated with resistance, including management practices, acaricide use frequency, and co-infestation with *R. microplus*. Although based on a limited number of farms, this represents the first assessment of acaricide resistance in the *A. cajennense* complex in Ecuador, as an initial resistance profile, these findings may provide a baseline for management *A. cajennense* in co-infestation with *R. microplus* and offer valuable insights for the development of more sustainable tick control strategies in Ecuador.

## 2 Methods

### 2.1 Study design and area

**Figure 1:**
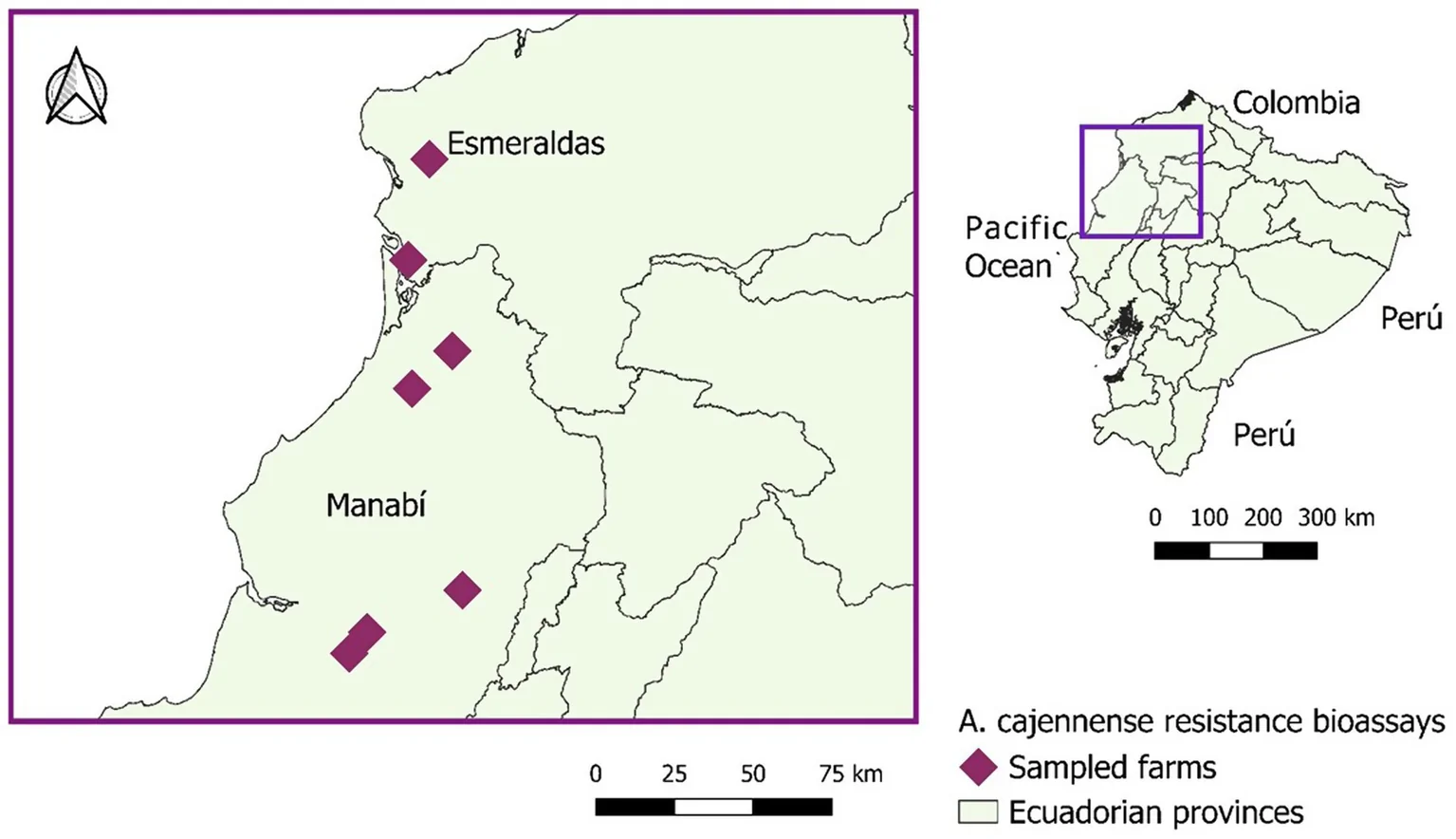
Farms sampled with *Amblyomma cajennense* s.l. presence and tested for acaricide resistance in Manabí and Esmeraldas.

A cross-sectional observational study was conducted to assess acaricide resistance in *A. cajennense* s.l. on cattle farms in Ecuador. Farms were identified using snowball sampling with the support of community leaders and veterinary technicians. Between May and August 2017, 97 cattle farms within ±0.5° of latitude were visited. Of these, 96 were infested with *R. microplus* resistance data reported elsewhere (15), one exclusively with *A. cajennense* s.l., and 17 with both species. Resistance testing requires at least 30 engorged females per farm. However, *A. cajennense* s.l. is more difficult to rear in the laboratory than *R. microplus*, and only seven farms (Esmeraldas, *n* = 2; Manabí, *n* = 5) met this criterion. Locations were georeferenced using a GPSmap64 Garmin^®^ device (WGS84, UTM 17S) (Figure 1). An epidemiological questionnaire was administered to farm owners by trained personnel to document cattle and farm management, tick infestation level, and tick control methods.

### 2.2 Tick collection, identification, and infestation assessment

**Figure 2:**
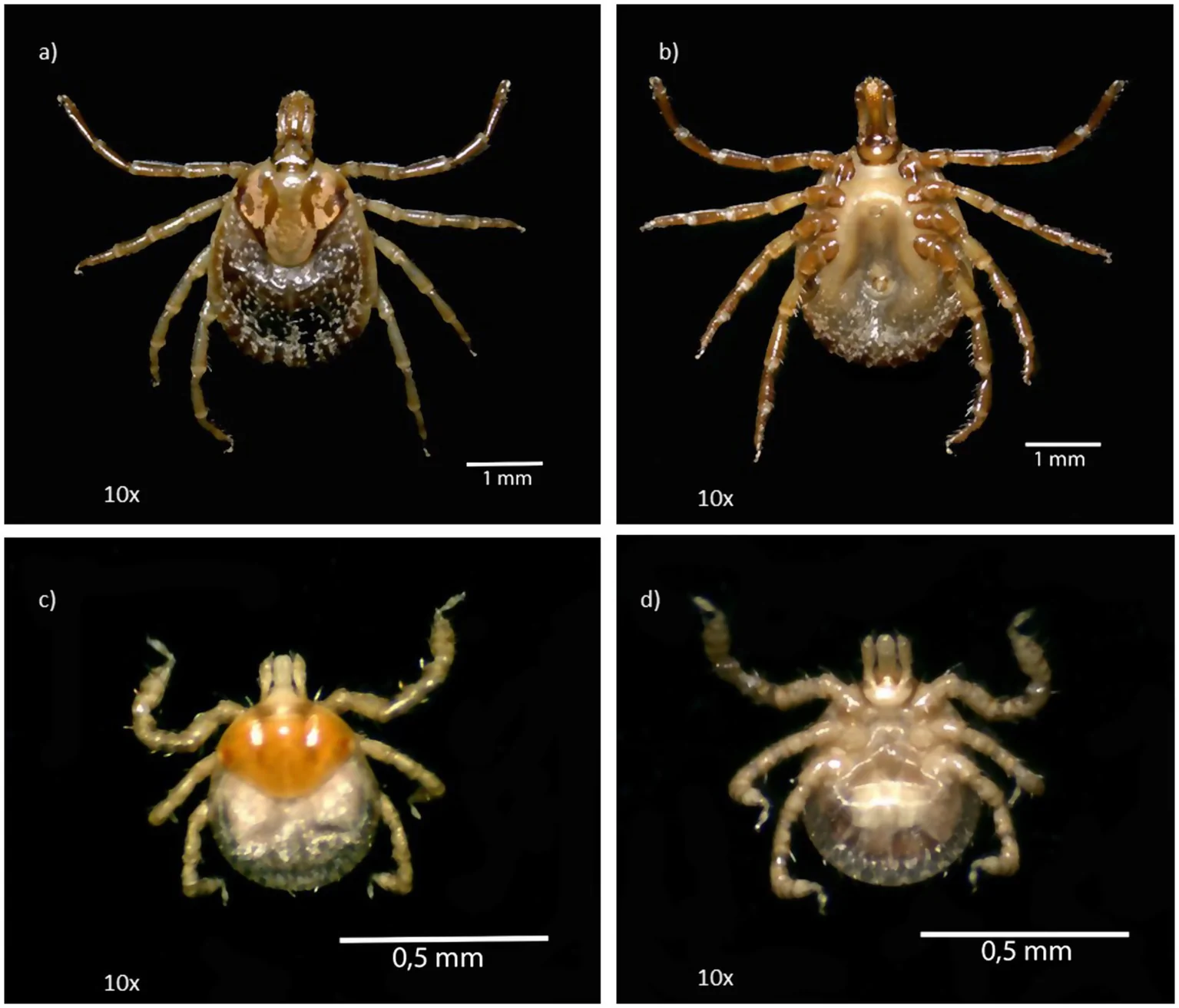
*Amblyomma cajennense* s.l. **(a)** Female adult specimen dorsal. **(b)** Female adult specimen ventral. **(c)** Larvae specimen dorsal. **(d)** Larvae specimen ventral. Scale 10x.

Ticks were collected from five randomly selected cattle per farm, targeting approximately 30 engorged female ticks per farm (11, 13, 16, 17). Specimens were kept in moistened containers and transported to the Entomology Laboratory of the Instituto de Investigación en Zoonosis (CIZ), Universidad Central del Ecuador. Morphological identification of *A. cajennense* s.l. was performed using dichotomous keys (7, 18) (Figure 2). Given the taxonomic complexity of the *A. cajennense* complex and the absence of molecular confirmation, we conservatively classified all specimens as *A. cajennense sensu lato*.

Tick infestation levels were assessed semi-quantitatively on the left side of each animal by the same trained observer. Only ticks >0.5 cm were counted. Low infestation was defined as no ticks or <20 ticks in ≤1 of three body regions; high infestation as >20 ticks in ≥2 regions. This approach is standard for tick burden assessment in Ecuador (12, 14).

### 2.3 Preparation of acaricide solutions and larval package test

Pure active ingredients of amitraz, ivermectin, and alpha-cypermethrin were obtained from a certified pharmaceutical company. Stock solutions were prepared following the manufacturer’s recommendations: amitraz and alpha-cypermethrin diluted in xylene (10 mL), and ivermectin in glycerin (w/v). These were stored at 100× concentration for up to 6 months (12). Working solutions were protected from direct sunlight and humidity. The concentrations levels used for dose–response assays were taken from Pérez-Otáñez et al. (15), with minimum, medium (discriminatory), and maximum doses as follows: amitraz at 0.025, 0.1, and 0.4%; alpha-cypermethrin at 0.005, 0.02, and 0.08%; and ivermectin at 0.025, 0.1, and 0.4%. The discriminatory dose corresponded to the concentration of commercial products sold in Ecuador.

Larval package tests (LPT) were used to evaluate the susceptibility or resistance of *A. cajennense* s.l. larvae (14–21 days old, 100 per package) to three acaricides. Filter papers (Whatman 541 type) (19) were impregnated with acaricide dilutions: ivermectin and alpha-cypermethrin were diluted in chloroform:olive oil (2:1), while amitraz required an additional nylon fabric base, (type 2,320 Cerex) (12, 16, 19). Packages were incubated at 29 °C and relative humidity of 80–90%. Mortality was assessed after 24 h for alpha-cypermethrin and ivermectin, and after 48 h for amitraz. Once the incubation period was completed, the packages were opened, and the number of alive and dead larvae was recorded (20–23). All concentrations were tested in duplicate, with a control per farm.

### 2.4 Data analysis

Resistance levels were assessed based on the results obtained at the discriminatory dose of each acaricide. Classification of resistance levels in the LPT followed the criteria outlined by Pérez-Otáñez et al. (15) (Table 1).

**Table 1:** Thresholds for farm-scale acaricide resistance identified through larval package test.

Category	Binary category	Larval survival %^a^
Susceptible	Not resistant	<10%
Low resistance	Resistant	11–20%
Medium resistance		21–50%
High resistance		>51%

^a^100 minus the corrected mortality.

Tick resistance level to acaricides on each farm was determined by calculating the corrected mortality rate for the LPT, as described in Rodríguez-Hidalgo et al. (12) and Abbott (24).


$$\begin{array}{l} \text{Corrected death rate for}\;\mathrm{LPT}\\ =\frac{(\text{mortality rate in the test}-\text{mortality rate of control group})\;x\;100}{100-\%\text{death rate of the control group}} \end{array}$$

The overall prevalence of resistant farms was calculated as the ratio of resistant farms to the total number of sampled farms (20).

Probit analysis (25) was used to estimate lethal doses (LD50 and LD99). Due to the unavailability of a reference control strain in Ecuador, discriminatory dose thresholds (low and high) were applied for comparative assessment. This method linearizes the dose–response relationship by converting mortality data into a probit scale, enabling precise modeling of the compound’s efficacy.

An exploratory univariable screening of management-related variables was performed to evaluate resistance in the different levels of the factors evaluated (Table 2).

**Table 2:** Variables obtained from an epidemiological questionnaire in each participating farm.

Variable	Description	Categories or units
Farm description		
Farm size	Heads of cattle on the farm	Large: 101–200 animals
		Medium: 51–100 animals
		Small: ≤ 50 animals
Production purpose	Production objective	Milk
		Dual-purpose
Management type	Whether the farm is managed extensively or intensively	Extensive
		Intensive
Stocking density	Number of cattle/ha	≤ 1.48 animal/ha
		>1.49 animal/ha
Silvopastoral system	If a forestry and grazing system is applied or not into the farm	Yes
		No
Animal management		
Bovine breed	Predominant breed into the farm	*Bos primigenius taurus*
		*Bos primigenius indicus*
Tick infestation degree*	Observational variable	High
		Low
Veterinary assistance	Frequence of visit of a veterinarian	Never and almost never
		Sometimes or permanent
Horse presence	If cattle share paddocks with horses	Yes
		No
Tick infestation management		
Chemical acaricide	If they applied chemical acaricide as the main control tick strategy or not	Yes
		No
Application per year	Number of acaricide applications per year	26, 52
Number of acaricides	Number of different active principles per year	1,2,3,4
For each acaricide under resistance study		
Acaricide used	Use of the amitraz, ivermectin 1%, or pyrethroids in the last year	Yes
		No
Correct dosage	Use of the correct dosage of the acaricide	Yes
		No
Frequency	Frequency of use for each acaricide	Each ≤ 2 months (High)
		Each > 2.1 months or not use (Low)

*Observational variable dividing the bovine into three parts, low = if there are no ticks or one third of the animal has more than 20 ticks, high = if two or three thirds of the animal have more than 20 ticks each.

### 2.5 Ethical considerations

Sample collection was supervised by licensed veterinarians in compliance with international animal welfare standards. Cattle were gently restrained using standard handling facilities (chutes or squeeze chutes) to minimize stress during tick collection. Ticks were collected manually by trained personnel using forceps, a non-invasive method that does not cause pain, injury, or distress to the animals. No animals were sedated or subjected to invasive procedures. Farmers received detailed explanations of study procedures and provided informed consent prior to participation. According to Ecuadorian regulations, this type of observational study involving non-invasive sampling of ectoparasites does not require formal ethical approval from a research ethics committee.

## 3 Results

### 3.1 Survey and farms visited

Of the farms visited, seven had sufficient engorged females for inclusion. All reported using chemical acaricides as their primary control method, though none used them at recommended doses. Treatment frequency was weekly on 57% (4/7) of farms and biweekly on 43% (3/7). Co-existence of *R. microplus* and *A. cajennense* s.l. occurred on 86% (6/7) of farms. Most farms (86%, 6/7) operated under extensive grazing systems, with one (14%) using intensive management. Indicine-adapted breeds were raised on 71% (5/7) of farms, taurine breeds on 29% (2/7). Equines were present on 71% (5/7) of farms. Most respondents (86%, 6/7) identified summer as the peak infestation season. None reported cattle deaths associated with hematuria.

### 3.2 Prevalence of resistance at the farm level

**Figure 3:**
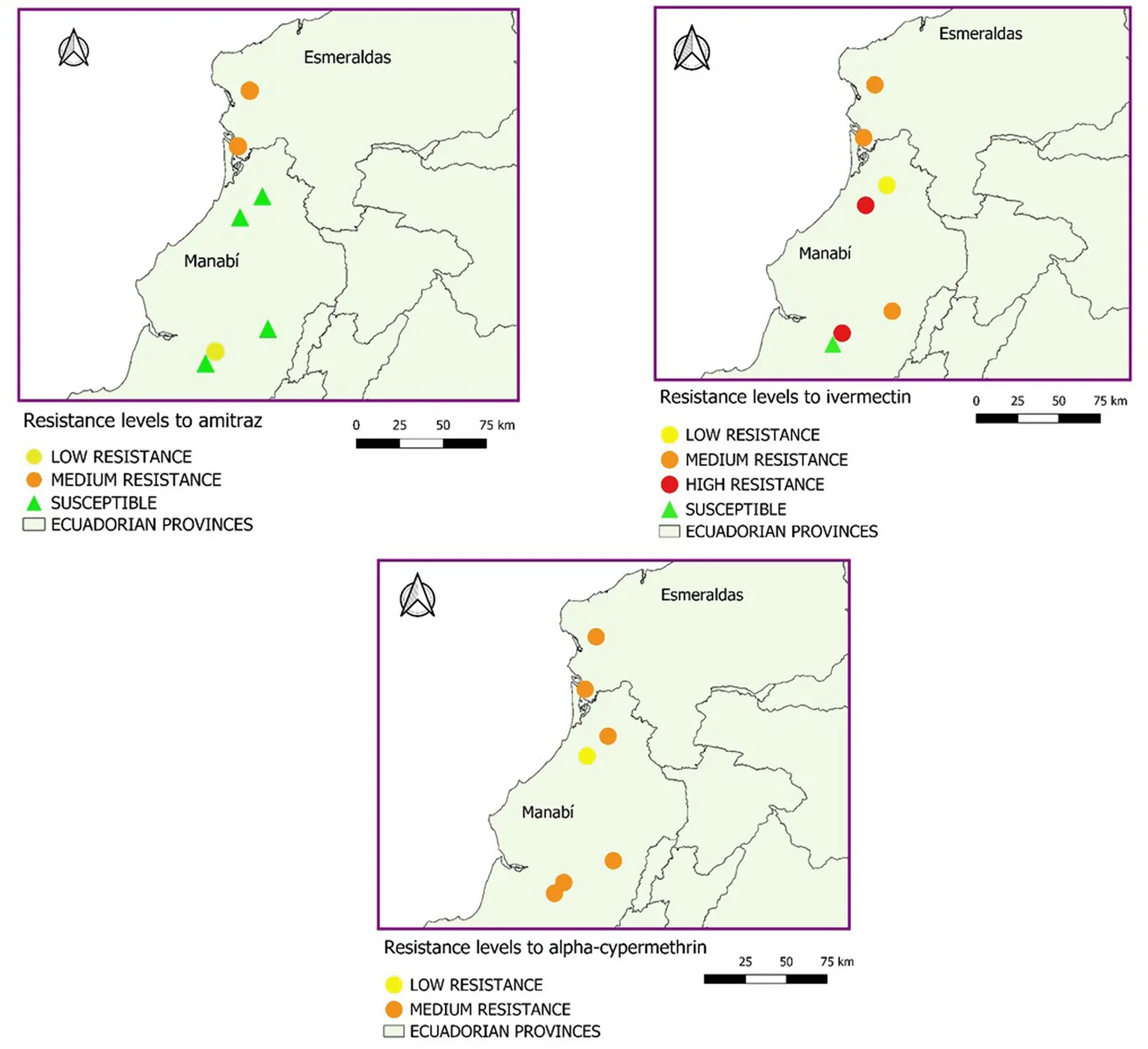
Farms with tick populations of *Amblyomma cajennense* sl.l. resistant and susceptible to amitraz, ivermectin and alphacypermethrin.

Resistance levels to the three acaricides varied across farms (Table 3). For amitraz, 57% (4/7) of farms were susceptible, while 43% (3/7) showed low (14%, 1/7) or medium resistance (29%, 2/7); no high resistance was detected. Ivermectin susceptibility was low (14%, 1/7), with resistance distributed as low (14%, 1/7), medium (43%, 3/7), and high (29%, 2/7). Alpha-cypermethrin showed no susceptibility; resistance was predominantly medium (86%, 6/7), with low resistance in the remaining farm (14%, 1/7) (Figure 3; Table 3). Overall, all farms (100%, 7/7) were resistant to at least one acaricide, and 86% (6/7) showed multi-resistance to two or more compounds.

**Table 3:** Resistance levels by larval package test in *Amblyomma cajennense* s.l.

Acaricide	Susceptible (0–10%)		Low resistance (11–20%)		Medium resistance (21–50%)		High resistance (>50%)	
	%	n/N	%	n/N	%	*n*	%	N
Amitraz	57.14	4/7	14.29	1/7	28.57	2/7	0.00	0/7
Ivermectin	14.29	1/7	14.29	1/7	42.86	3/7	28.57	2/7
Alpha-cypermethrin	0.00	0/7	14.29	1/7	85.71	6/7	0.00	0/7

Table 4 presents the proportion between farm management factors and the presence of acaricide resistance. Among the acaricides evaluated, amitraz showed the lowest level of resistance; however, farms operating under extensive production systems exhibited higher resistance levels than intensive systems (50% vs. 0%). Similarly, the presence of alternative tick hosts, such as horses, appeared to be associated with increased resistance levels (60% vs. 0%). In contrast, ivermectin showed high levels of tick resistance across farms, and patterns similar to those observed for amitraz were detected, with higher resistance in extensive systems and in farms where alternative hosts were present. For alpha-cypermethrin, none of the evaluated management factors showed clear differences between resistance levels; however, this acaricide exhibited consistently high resistance across all farm-characterization categories (86%).

**Table 4:** Results of proportion of high level of tick-acaricide resistance with variables obtained from the survey and the resistant status from LPT.

Variable	Levels	Farms	Amitraz		Ivermectin		Alpha-cypermethrin	
			Resistance farms	% resistance	Resistance farms	% resistance	Resistance farms	% resistance
Farm description	Large: 101–200 animals	2	1	50%	2	100%	2	100%
Farm size	Medium: 51–100 animals,	3	1	33%	3	100%	3	100%
	Small: ≤ 50 animals	2	1	50%	1	50%	2	100%
Production purpose	Dual-purpose	7	3	43%	6	86%	7	100%
Management type	Extensive	6	3	50%	6	100%	6	100%
	Intensive	1	0	0%	0	0%	1	100%
Stocking density	≤ 1.48 animal/ha	7	3	43%	6	86%	7	100%
Silvopastorail system	Yes	5	3	60%	4	80%	5	100%
	No	2	0	0%	2	100%	2	100%
Bovine breed	*Bos primigenius taurus*	2	1	50%	2	100%	2	100%
	*Bos primigenius indicus*	5	2	40%	4	80%	5	100%
Tick infestation	Low	7	3	43%	6	86%	7	100%
Veterinary	No (never and almost never)	7	3	43%	6	86%	7	100%
Horses presence	Yes	5	3	60%	5	100%	5	100%
	No	2	0	0%	1	50%	2	100%
Chemical acaricide	Yes	7	3	43%	6	86%	7	100%
Acaricide used	Yes	4	3	75%	4	100%	4	100%
	No	3	0	0%	2	67%	3	100%
Correct dosage	No	7	3	43%	6	86%	7	100%
Frequency	Each ≤ 2 months (High)	3	0	0%	2	67%	3	100%
	Each > 2.1 months or not use (Low)	4	3	75%	4	100%	4	100%

### 3.3 Lethal doses

Lethal dose (LD) values for amitraz, ivermectin, and alpha-cypermethrin against *A. cajennense* s.l. populations are shown in Table 5. Amitraz had an LD₅₀ of 0.001% (95% CI: 0–0.002%), with LD₉₀ and LD₉₉ increasing to 0.64% (0.27–1.57%) and 134.52% (55.27–327.41%), respectively. Ivermectin required higher concentrations: LD₅₀ = 0.03% (0.02–0.05%), LD₉₀ = 12.56% (8.40–18.80%), and LD₉₉ = 1571.49% (1050.10–2351.80%). Alpha-cypermethrin showed an LD₅₀ similar to amitraz (0.001%; 0–0.002%) but markedly higher LD₉₀ (3.26%; 1.57–6.79%) and LD₉₉ (3019.78%; 1450.47–6286.95%) values, the highest among the three acaricides. Overall, all compounds showed a progressive increase in the dose required to reach higher mortality thresholds (90 and 99%), with estimated values substantially exceeding recommended commercial concentrations. Confidence intervals reflect variability in the response among tested populations (Table 5).

**Table 5:** Lethal doses obtained from probit analysis with the mortality data from LPT in *A. cajennense* s.l. population ticks.

Active ingredient	%LD 50 (95% lower-upper)	% LD 90 (95% lower-upper)	% LD 99 (95% lower-upper)	Discriminatory dose %
Amitraz	0.001 (0–0.002)	0.64 (0.27–1.57)	134.52 (55.27–327.41)	0.1
Ivermectin	0.03 (0.02–0.05)	12.56 (8.40–18.80)	1571.49 (1050.10–2351.80)	0.1
Alpha-cypermethrin	0.001 (0–0.002)	3.26 (1.57–6.79)	3019.78 (1450.47–6286.95)	0.02
LD = lethal dosses				

## 4 Discussion

### 4.1 Key findings

This study provides the first evidence of acaricide resistance in *A. cajennense* s.l. in Ecuadorian cattle farms. Our bioassays across seven farms revealed concerning resistance trends, particularly where both species coexist. Amitraz resistance was 43%, ivermectin resistance was 86%, and alpha-cypermethrin resistance was 100%. For all three acaricides, LD₉₉ values substantially exceeded maximum tested concentrations: amitraz by 336-fold (134.52% vs. 0.4%), ivermectin by 3,929-fold (1571.49% vs. 0.4%), and alpha-cypermethrin by 37,747-fold (3019.78% vs. 0.08%). Even at recommended doses, a small proportion of surviving ticks may sustain local populations.

### 4.2 Comparison with prior research

Our amitraz resistance (43%) is higher than the 12.5% reported in Mexico for *A. cajennense* (11), and contrasts with the limited amidine efficacy (24.4% against nymphs, no adult effect) reported for *A. mixtum* (26), suggesting variability within the *A. cajennense* complex.

No prior regional studies have analyzed macrocyclic lactones in this species, making this the first evidence of ivermectin resistance in *A. cajennense* s.l. The high resistance level (86%) suggests intensive use of macrocyclic lactones or cross-resistance mechanisms, consistent with ivermectin resistance in *R. microplus* (42–70%) in Ecuador. Compared to *R. microplus*, our findings show higher resistance in *A. cajennense* s.l., suggesting species-specific vulnerabilities.

For alpha-cypermethrin, our findings (100%) contrast with susceptibility to other pyrethroids reported by Alonso-Díaz et al. (11), but align with Higa et al. (26) who reported very low cypermethrin efficacy (2.2%) against *A. mixtum*. In Ecuador, pyrethroid resistance in *R. microplus* is widespread (64–68% of populations), suggesting cross-resistance and sustained selection pressure (27).

Historical efficacy studies date back to 1977, reporting higher LD₅₀ values for *A. cajennense* than other species (28), contrasting with Drummond (29) who found acaricides effective against five tick species also worked against *A. cajennense*. Our findings align with previous reports showing that *A. cajennense* s.l. tolerates higher acaricide concentrations than *R. microplus* (30, 31). These authors recommended doses at least 1.8 times higher for *A. cajennense* than for *R. microplus*. This inherent tolerance reinforces the need for species-specific resistance reporting, as data from *R. microplus* cannot be directly extrapolated to *A. cajennense* (19).

### 4.3 Integration and advancement of current understanding

Although *R. microplus* remains the predominant species nationally, *A. cajennense* s.l. has a coastal-specific distribution driven by bioclimatic preferences (32), emphasizing the need for geographically tailored control. The distinct biology of *A. cajennense* s.l., broader host range, longer off-host survival, three-host life cycle, complicates control and limits one-size-fits-all approaches (33, 34). Conventional measures targeting *R. microplus* (one-host) have limited impact on *Amblyomma* spp. populations maintained by alternative hosts and environmental stages. Co-infestation (86% of farms) likely exacerbates resistance, as acaricides are indiscriminately applied, promoting cross-resistance (35–38). In Ecuador, chemical acaricides remain the primary control method, with documented *R. microplus* resistance highlighting the urgency of evaluating other species (6, 12).

These findings have practical implications for tick control in Ecuador. First, alpha-cypermethrin should no longer be used as a first-line acaricide against *A. cajennense* s.l. in coastal Ecuador, given the 100% resistance prevalence. However, we acknowledge that farmers may have limited access to alternative compounds due to cost or availability. In such cases, we recommend restricting alpha-cypermethrin use to rotational schemes with efficacy testing, rather than complete withdrawal, as a transitional measure. Second, reliance on macrocyclic lactones for tick-specific control should be reconsidered given the high ivermectin resistance (72%); its use should be reserved for endoparasite control to preserve its efficacy. Third, amitraz requires susceptibility testing before use, as resistance is emerging (43%). Given the three-host biology of *A. cajennense* s.l., acaricide rotation alone is insufficient; integrated strategies must include pasture management (e.g., rotational grazing to interrupt off-host life stages) (8), host-targeted treatments (e.g., treating equines which act as reservoirs) (39), and reduced treatment frequency (19). We also recommend long-term resistance monitoring to evaluate mitigation effectiveness, alongside education and awareness campaigns targeting farmers and veterinary technicians in the region.

### 4.4 Limitations

Methodological challenges include the limited sample size (seven farms) and the use of snowball sampling, which may introduce selection bias, as farms were identified through community leaders and veterinary technicians rather than randomly selected. This approach likely favored farms with perceived tick problems or known acaricide failure, potentially overestimating resistance prevalence. Consequently, our findings should be interpreted as indicative rather than representative of the broader *A. cajennense* s.l. population in Ecuador. However, despite these limitations, this study represents the first report of acaricide resistance in *A. cajennense* s.l. in Ecuador. Without this initial evidence, the potential for resistance in this species would remain undocumented, and the need for larger, more comprehensive studies would lack a scientific basis. Thus, while our results are preliminary, they provide a critical foundation for future research and highlight the importance of including *A. cajennense* s.l. in resistance surveillance programs. Additionally, the absence of a reference susceptible strain prevented full susceptibility baseline comparisons. We could not identify risk factors associated with resistance due to the small sample.

### 4.5 Future directions

Until larger, more representative datasets are available, resistance mitigation should rely on precautionary principles: proactive monitoring and adaptive management. Emerging alternatives such as plant-derived compounds and bacterial formulations have shown promise *in vitro* (40–42), but their implementation requires prior knowledge of local resistance profiles. We also need education, training, and awareness campaigns for farmers and veterinary technicians to promote rational use of acaricides.

### 4.6 Postulated theories

Based on our findings, we propose two testable hypotheses for future research. First, the extreme LD₉₉ values for alpha-cypermethrin (37,747-fold above field doses) may reflect constitutive detoxification mechanisms in *A. cajennense* s.l. rather than acquired resistance (43). This could be tested by comparing baseline metabolic enzyme activity (e.g., esterases, cytochrome P450s) between *A. cajennense* and *R. microplus* populations with no prior acaricide exposure (44). Second, horses and other alternative hosts act as untreated reservoirs, maintaining resistant *A. cajennense* populations despite cattle treatments (1, 45). This can be tested by comparing resistance allele frequencies in ticks collected from cattle versus horses on the same farm. If confirmed, control strategies should explicitly include alternative hosts rather than focusing solely on cattle. High ivermectin usage is explained by its broad-spectrum activity against internal and external parasites (46). Extensive husbandry systems may promote reduced or improperly applied acaricide doses, further contributing to resistance development.

## 5 Conclusion

This study provides the first evidence of acaricide resistance in *Amblyomma cajennense* s.l. populations from cattle farms in Ecuador. Resistance was detected for all evaluated compounds, with moderate levels for amitraz but markedly higher levels for ivermectin and alpha-cypermethrin. The extremely high LD₉₀ and LD₉₉ values, frequently exceeding recommended commercial concentrations by several orders of magnitude, indicate reduced susceptibility and suggest limited field efficacy of these compounds against some populations. Exploratory, qualitative screening of farm management factors suggested that extensive production systems and the presence of alternative hosts, particularly horses and other equids, may contribute to the development and persistence of resistance, potentially through reduced or inconsistent acaricide application and untreated reservoir hosts. These findings are particularly relevant because *A. cajennense* s.l. frequently co-occurs with *R. microplus* on cattle farms, although the two species differ markedly in ecology, host range, and life cycle. Control strategies designed primarily for *R. microplus* may have limited impact on *Amblyomma* populations maintained through wildlife and environmental stages. Under these conditions, the emergence of acaricide resistance, especially the high levels observed for alpha-cypermethrin, may further compromise integrated tick control and highlights the need for species-specific management strategies and expanded surveillance programs in Ecuador.

## Data Availability

The raw data supporting the conclusions of this article will be made available by the authors, without undue reservation.
